# The Impact of the Ongoing COVID-19 Epidemic on the Increasing Risk of Adverse Pathology in Prostate Cancer Patients Undergoing Radical Prostatectomy

**DOI:** 10.3390/curroncol29040225

**Published:** 2022-04-15

**Authors:** Łukasz Nyk, Hubert Kamecki, Bartłomiej Zagożdżon, Andrzej Tokarczyk, Piotr Baranek, Łukasz Mielczarek, Piotr Kryst, Sławomir Poletajew, Roman Sosnowski, Stanisław Szempliński

**Affiliations:** 1 Second Department of Urology, Centre of Postgraduate Medical Education, 01-809 Warsaw, Poland; lukasz.nyk@cmkp.edu.pl (Ł.N.); bartlomiej.zagozdzon@ecz-otwock.pl (B.Z.); andrzej.tokarczyk@ecz-otwock.pl (A.T.); piotr.baranek@ecz-otwock.pl (P.B.); lukasz.mielczarek@ecz-otwock.pl (Ł.M.); piotr.kryst@bielanski.med.pl (P.K.); slawomir.poletajew@cmkp.edu.pl (S.P.); stanislaw.szemplinski@cmkp.edu.pl (S.S.); 2 Department of Urogenital Cancer, Maria Skłodowska-Curie National Research Institute of Oncology, 02-781 Warsaw, Poland; roman.sosnowski@gmail.com

**Keywords:** prostatic neoplasms, COVID-19, biopsy, adverse pathology, prostatectomy

## Abstract

We aimed to assess whether the ongoing course of the COVID-19 epidemic has been associated with an increased risk of adverse pathology (AP) findings in prostate cancer (PC) patients treated with radical prostatectomy (RP). We performed a retrospective data analysis which included 408 consecutive, non-metastatic, previously untreated PC patients who underwent RP in our institution between March 2020 and September 2021. Patients were divided into two equally numbered groups in regard to the median surgery date (Early Epidemic [EE] and Late Epidemic [LE]) and compared. Adverse pathology was defined as either grade group (GG) ≥ 4, pT ≥ 3a or pN+ at RP. Patients in the LE group demonstrated significantly higher rates of AP than in the EE group (61 vs. 43% overall and 50 vs. 27% in preoperative non-high-risk subgroup, both *p* < 0.001), mainly due to higher rates of upgrading. On multivariable analysis, consecutive epidemic week (odds ratio: 1.02, 95% confidence interval: 1.00–1.03, *p* = 0.009) as well as biopsy GG ≥ 2 and a larger prostate volume (mL) were associated with AP in non-high-risk patients. The study serves as a warning call for increased awareness of risk underassessment in contemporarily treated PC patients.

## 1. Introduction

For the past two years, the ongoing COVID-19 pandemic has been posing a significant and an unprecedented challenge to providing adequate healthcare to patients with suspected or diagnosed urologic malignancies. Several strategies to overcome the crisis have been proposed, including procedure triaging [1] and maintaining care via means of telemedicine [2]. Any potential long-term effects that may have been induced to urologic cancer management by lockdowns and distancing measures are yet to be evaluated. However, it is possible that the actual impact was significantly negative.

The first COVID-19 case in Poland was identified in March 2020 [3]. Since that time, many healthcare lockdown measures have been imposed in the country, including telehealth implementation, periodical transforming of wards into infectious disease units, and limiting hospital admissions for planned procedures. The geographic distribution of these actions was heterogenic, as well as as variable in time, and while there have been efforts to investigate a possible deterioration in the quality of medical care in Poland [4], no systematic analysis of healthcare maintenance during the COVID-19 epidemic in the country has been carried out to date.

In this study, we aimed to assess the short-term effect of the ongoing COVID-19 epidemic in Poland on the treatment results of prostate cancer patients undergoing radical prostatectomy in our institution, by investigating for possible associations between the ongoing course of the epidemic and the risk of adverse final pathology findings.

## 2. Materials and Methods

We retrospectively analyzed the data of consecutive prostate cancer (PC) patients who had undergone radical prostatectomy (RP) in our institution between March 2020 and September 2021. The reason for March 2020 being set as the lower date limit was the fact that this was the first month of COVID-19 cases having been identified in Poland [3]; September 2021 was the month of data having been collected.

The patient inclusion criteria were as follows: (i) having undergone RP for non-metastatic PC, (ii) having undergone preoperative or pre-biopsy multiparametric magnetic resonance imaging (mpMRI) of the prostate, and (iii) no history of other previous treatment for PC. Patients with missing data in regard to: (i) preoperative prostate-specific antigen (PSA) level, (ii) tumor stage at mpMRI, (iii) biopsy grade group, and (iv) RP pathology report were excluded from the study.

Data was collected from the electronic patient records and included: age, preoperative PSA level, data on the preoperative mpMRI and preoperative prostate biopsy, date of surgery, and an RP pathology report.

Due to the non-interventional character of the study and data having been collected anonymously, an ethics board approval was not required for this study, which was in accordance with state law regulations.

### 2.1. Definitions

We defined preoperative high-risk as any of the following: (i) preoperative PSA level ≥ 20 ng/mL, (ii) extra-prostatic extension (EPE) or seminal vesicle invasion (SVI) reported on mpMRI, or (iii) grade group ≥ 4 at biopsy. Adverse pathology was defined as any of the following: (i) grade group ≥ 4, (ii) tumor stage pT3a or higher, or (iii) lymph node involvement (LNI) at final (RP) pathology.

Epidemic day was the date of surgery, expressed as the number of days that had passed since 1 March 2020; epidemic week was calculated by dividing the epidemic day by 7.

### 2.2. Outcome Measurements and Statistical Analysis

Patients were divided into two groups:

1. The Early Epidemic (EE) group, for the first half of patients who underwent RP between 2020 and 2021, as sorted in ascending chronological order by the surgery date,
2. The Late Epidemic (LE) group, for the second half of patients.

The two groups of patients were compared in regard to demographics, preoperative and postoperative clinical characteristics, with subsequent analyses limited to non-high-risk patients. Mann–Whitney and Chi-square tests were used to compare continuous variables and percentages, respectively.

Subsequently, we identified all non-high-risk patients within the EE and LE groups to create a cohort that underwent further analyses aimed at determining the risk factors for adverse pathology during the ongoing pandemic. We investigated the associations between adverse pathology, date of surgery (epidemic week), and preoperative clinical characteristics. The associations of continuous and categorical variables with the dependent variable were first investigated with univariable logistic regression. The variables that demonstrated a trend towards a significant association (*p* value < 0.15) were then included in a multivariable logistic regression model.

The outcomes of analyses were expressed as odds ratios (ORs) with 95% confidence intervals (95% CIs). We considered the observed outcomes statistically significant when *p*-value < 0.05. Continuous and categorical variables were expressed as median (with interquartile ranges) and numbers (with percentages), respectively.

Statistical analyses were performed using PSPP (GNU, version 1.4.1-g79ad47, Free Software Foundation, Boston, MA, USA).

## 3. Results

We identified 458 patients who underwent RP and met the inclusion criteria. Of these, 50 patients were excluded: 36 due to incomplete mpMRI staging, 12 due to incomplete final pathology reports, 1 due to a missing preoperative PSA level, and 1 due to missing data in regard to biopsy results. Finally, 408 men were included in the study.

The 408 included patients who underwent surgery between March 2020 and September 2021 were sorted in ascending chronological order by the surgery date. The median surgery date was 24 November 2020. The patients were divided into 2 groups of 204 consecutive patients: the EE and the LE group.

The comparison of patients in the EE and the LE groups in regard to demographic, preoperative, and postoperative clinical characteristics is presented in Table 1. The analysis limited to non-high-risk patients is presented in Table 2.

The results of both uni- and multivariable analyses aimed to determine the association between the date of surgery, as well as specific patient- or disease-related factors and the risk of adverse pathology within the cohort of non-high-risk patients who underwent RP between March 2020 and September 2021 is presented in Table 3.

## 4. Discussion

In our study we demonstrated that the date of surgery, or the count of time that had passed since the onset of the COVID-19 epidemic in Poland, was a significant factor associated with adverse pathology in prostate cancer patients who underwent radical prostatectomy in our institution, most importantly in men preoperatively deemed to harbor non-high-risk disease. To our knowledge, this is the first study to assess the impact of the ongoing course of the epidemic on postoperative outcomes in men treated for prostate cancer.

A recent multicenter retrospective study by Zattoni et al. [5] demonstrated that PC patients treated with RP during the epidemic had higher rates of EPE or LNI at final pathology in a year-over-year comparison to an earlier period. The study did not, however, investigate for an association between a change in pathology outcomes and the ongoing time of the epidemic, which would be of clinical significance considering the slow natural history of prostate cancer and that effects of any disruptions in diagnosis and early management would be expected to be observed with a delay. In our study, we demonstrated that while patients who underwent RP later in the course of the epidemic—in comparison to patients treated in the earlier months—did not significantly differ in regard to their preoperative risk profile, while they did differ in regard to final pathology results.

An intuitive, possible explanation of our findings would be that various negative effects imposed by the COVID-19 epidemic-related lockdowns or limitations could have led to a deterioration in healthcare or limited access to medical services, hence resulting in an increased risk of disease progression among PC patients awaiting prompt and proper management. For example, Coma et al. demonstrated that the lockdown in Catalonia led to a significant reduction in prostate cancer incidence in comparison to a period before the lockdown, with baseline levels having not been reached since [6]. We lack access to similar data in regard to Poland, but one may suspect that continuity or quality of care at multiple stages of prostate cancer diagnosis and management may have been negatively impacted by epidemic-related issues. However, considering that studies on large retrospective cohorts demonstrated no association between a delay in RP and a risk of adverse final pathology results [7,8], deferrals or postponements of treatment, if any, can be deemed unlikely to represent the underlying cause of impaired outcomes in our patients. However, aside from biopsy-to-RP time, it is worth mentioning that no study has evaluated a possible association between adverse pathology and a prolonged time between first diagnostic procedures, e.g., first elevated PSA level or first suspicious digital rectal examination, and RP, in the context of COVID-19 epidemic.

Another possible explanation of our findings would be that epidemic-related issues were responsible for an increased risk of incorrect preoperative risk assessment. Assuming that some of the non-high-risk patients in the Late Epidemic group would have been preoperatively recognized as high-risk if they had not been understaged or undergraded, the observed differences in adverse final pathology rates should not have demonstrated statistical significance if the patients had been accurately assessed. In the analysis performed in the subgroup of non-high-risk patients in whom adverse pathology at RP may be considered as evidence of preoperative risk underassessment, two of the factors that comprise for AP according to the definition adopted for this study, namely: grade group ≥ 4 and LNI, were reported significantly more frequently in the LE than in the EE group.

Despite no statistically significant differences in regard to particular grade group rates at biopsy between LE and EE groups, grade group ≥ 4 cancer at final pathology was being reported more frequently in the LE group. Although a possible link between COVID-19 and cancer progression has been recently discussed in the literature [9], we consider it unlikely that the ongoing course of the epidemic caused grade progression in our patients. Undergrading at biopsy appears to be the most reasonable cause of these findings. Kaufman et al. have recently reported decreasing rates of grade group 4 cancers being diagnosed at biopsy during the COVID-19 pandemic [10]; the question of whether this was caused by undergrading, remains unanswered. It has been raised in the literature that the risk of SARS-CoV-2 transmission may be increased during a prostate biopsy procedure [11]. While we lack data in regard to factors that might have influenced the quality of prostate biopsy in our patients, e.g., number of cores taken or total core length, we cannot exclude that this quality has been compromised in the course of the epidemic, possibly due to social distancing measures or fear of transmission.

The other adverse finding reported at higher rates in the LE group in non-high-risk patients was LNI. However, it is hard to discuss possible underassessment in regard to this matter. Preoperative predicting of LNI in PC patients is challenging. Apart from additional imaging studies—which are of limited sensitivity [12] and not routinely recommended in every man prior to RP, especially in the non-high-risk subpopulation [13]—nomograms have been developed and implemented for the purpose of planning lymphadenectomy extent [14]. Unfortunately, we lack data in regard to calculated preoperative predictions of LNI probability in our patients. However, as the contemporarily used nomograms incorporate baseline disease characteristics that did not differ between the patient groups, most probably the preoperative LNI probability predictions were not significantly different as well. The most reasonable explanation for a higher rate of LNI in the LE group is the higher rate of grade group ≥4 cancer. Higher grade group patients were more likely to develop lymph node metastases, which is a known feature of the natural history of prostate cancer [15].

Being aware that there may have been factors other than the ongoing course of the epidemic that contributed to the occurrence of adverse pathology findings at RP, we performed further analyses to study for possible interdependencies between several baseline patient or disease characteristics and the risk of AP in non-high-risk patients. We included age in the analyses, as older men have been reported to harbor AP more frequently, especially in low-risk PC populations [16,17]; however, we found no significant association in our patient group. We demonstrated that grade group ≥2 was associated with an increased risk of AP, which is not surprising [18]. We arbitrarily decided to analyze prostate specific antigen (PSA) level and prostate volume (PV) separately, instead of calculating possible associations for PSA density (PSAD). With this method, we did not find a statistically significant relationship between a preoperative PSA level and the risk of AP. This is an intriguing finding, as higher PSA levels have been linked with an increased risk of AP both in low- and intermediate-risk PC [18,19]. A possible explanation is that the relatively low PSA levels among non-high-risk men in our study may not have been commonly reaching the levels at which serum PSA strongly correlates with AP. As higher PSAD is a known risk factor for AP [20], it is not surprising that PV demonstrated a negative association with AP in our patients. However, considering the fact that we did not find such an association for PSA, based on the results of the multivariable analysis we provide additional important evidence that lower prostate volume may be independently associated with a more aggressive course of prostate cancer [21,22].

On the multivariable analysis, which included the abovementioned factors, epidemic week was significantly associated with adverse pathology in non-high-risk patients who underwent RP during the course of the epidemic. This result further emphasizes the impact of the ongoing course of the epidemic on risk upgrading in our patients.

There are several limitations of our study, mainly related to its retrospective design. Firstly, access to additional data could have provided a deeper insight into the obtained results. Most importantly, any data necessary for the assessment of the quality of biopsies performed in our patients could have helped in evaluating for possible impact on preoperative undergrading and thus on the calculated associations. Also, biopsy-to-surgery times could allow for excluding the potential, yet neglected by the literature, effect of possible delay of surgery on pathologic outcomes. Secondly, we did not include patients treated before March 2020 and thus we lacked the opportunity to study the demonstrated time-dependent relationships within a longer period of time, which could have helped in excluding possible confounders that were not related to the epidemic. This was, however, intentional, as before 2020, which was the year we changed our institutional policy regarding the management of low-risk PC, many RPs in our institution were performed on low-risk patients and including this cohort would have posed a significant risk of selection bias. Another issue that one may consider a limitation to the study is that not all prostate biopsy specimens and mpMRI scans were performed or reviewed in our institution, as we serve as a tertiary referral center for patients initially diagnosed in other institutions. However, including these external results in our analyses better reflects the typical clinical scenario and provides more insight into the nation-wide healthcare disturbances, which was one of the aims of this study.

## 5. Conclusions

In our study, we demonstrated that the ongoing course of COVID-19 epidemic in Poland was associated with increasing rates of adverse pathology findings in patients undergoing radical prostatectomy for prostate cancer, which was of special significance in men preoperatively diagnosed with non-high-risk disease. While undergrading at biopsy had been a probable cause of the observed findings, further research should be aimed at determining the reasons for declined outcomes. Regardless of limitations, resulting mainly from the retrospective design, this study serves as a warning call for increased awareness of risk underassessment in contemporarily treated prostate cancer patients.

## Figures and Tables

**Table 1:** The comparison of patient groups.

		EE (*n* = 204)	LE (*n* = 204)	LE vs. EE *p*-Value
Median age, year [IQR]		65 [61–69]	66 [61–70]	0.186
Median PSA, ng/mL [IQR]		8.0 [5.5–13.0]	8.0 [5.4–12.7]	0.958
Grade group at biopsy	1	67 (33%)	54 (26%)	0.159
	2	84 (41%)	87 (43%)	0.763
	3	29 (14%)	31 (15%)	0.800
	4	16 (8%)	24 12%)	0.183
	5	8 (4%)	8 (4%)	1.000
Grade group ≥ 4 at biopsy		24 (12%)	32 (16%)	0.250
Highest PI-RADS-2 category	2	4 (2%)	6 (3%)	0.522
	3	17 (8%)	17 (8%)	1.000
	4	92 (45%)	92 (45%)	1.000
	5	83 (41%)	87 (43%)	0.688
	none ^a^	8 (4%)	2 (1%)	0.055
EPE or SVI at mpMRI		34 (17%)	41 (20%)	0.371
Prostate volume, mL [IQR]		39 [31–55]	38 [30–49]	0.319
Preoperative high-risk		62 (30%)	71 (35%)	0.342
Grade group at RP	1	23 (11%)	10 (5%)	**0.018**
	2	74 (36%)	70 (34%)	0.679
	3	67 (33%)	58 (28%)	0.334
	4	33 (16%)	50 (25%)	**0.037**
	5	7 (3%)	16 (8%)	0.053
Grade group ≥ 4 at RP		40 (20%)	66 (32%)	**0.003**
Stage at surgery	pT2	130 (64%)	124 (61%)	0.540
	pT3a	48 (24%)	52 (25%)	0.645
	pT3b/4	26 (13%)	28 (14%)	0.770
pT3a or higher at surgery		74 (26%)	80 (39%)	0.540
pN+ at surgery		21 (10%)	32 (16%)	0.105
Adverse pathology		87 (43%)	124 (61%)	**<0.001**

EE—Early Epidemic; LE—Late Epidemic; PSA—prostate-specific antigen; IQR—interquartile range; PI-RADS-2—Prostate Imaging-Reporting and Data System-2; EPE—extra-prostatic extension; SVI—seminal vesicle invasion; mpMRI—multiparametric magnetic resonance imaging; RP—radical prostatectomy. ^a^ a lesion non-identifiable on mpMRI or not reported according to PI-RADS-2 guidelines. Bold: *p* < 0.05.

**Table 2:** The comparison of patient groups limited to non-high risk patients only.

		EE (*n* = 142)	LE (*n* = 133)	LE vs. EE *p*-Value
Median age, year [IQR]		66 [61–69]	65 [60–70]	0.614
Median PSA, ng/mL [IQR]		7.2 [5.4–9.9]	7.1 [5.3–10.0]	0.967
Grade group at biopsy	1	58 (41%)	45 (34%)	0.230
	2	67 (47%)	70 (53%)	0.366
	3	17 (12%)	18 (14%)	0.698
Highest PI-RADS-2 category	2–3	15 (11%)	20 (15%)	0.266
	4–5	126 (89%)	112 (84%)	0.272
	none ^a^	1 (1%)	1 (1%)	0.963
Prostate volume, mL [IQR]		38 [29–54]	36 [30–47]	0.947
Grade group at RP	1	22 (15%)	10 (8%)	**0.039**
	2	63 (44%)	53 (40%)	0.449
	3	47 (33%)	43 (32%)	0.892
	4	10 (7%)	27 (20%)	**0.001**
	5	0 (0%)	0 (0%)	N/A
Grade group ≥ 4 at RP		10 (7%)	27 (20%)	**0.001**
Stage at surgery	pT2	110 (77%)	95 (71%)	0.251
	pT3a	29 (30%)	32 (24%)	0.468
	pT3b/4	3 (3%)	6 (5%)	0.264
pT3a or higher at surgery		32 (23%)	38 (29%)	0.251
pN+ at surgery		2 (1%)	10 (8%)	**0.013**
Adverse pathology		39 (27%)	66 (50%)	**<0.001**

EE—Early Epidemic; LE—Late Epidemic; PSA—prostate-specific antigen; IQR—interquartile range; PI-RADS-2—Prostate Imaging-Reporting and Data System-2; RP—radical prostatectomy. ^a^ a lesion non-identifiable on mpMRI or not reported according to PI-RADS-2 guidelines. Bold: *p* < 0.05.

**Table 3:** Associations between the date of surgery (epidemic week), patient- and disease-related variables, and the risk of adverse pathology within the cohort of non-high-risk patients.

		Adverse Pathology vs. No Adverse Pathology			
		UVA		MVA	
Variable		OR (95% CI)	*p*-Value	OR (95% CI)	*p*-Value
Epidemic week		1.02 (1.01–1.03)	**0.003**	1.02 (1.00–1.03)	**0.009**
Age, year		1.00 (0.97–1.04)	0.814	– ^a^	
PSA, ng/mL		1.06 (0.99–1.13)	0.118	1.07 (1.00–1.15)	0.067
Grade group at biopsy	≥2 ^b^	2.17 (1.28–3.68)	**0.004**	1.93 (1.10–3.37)	**0.021**
	≥3 ^c^	1.63 (0.80–3.33)	0.176	– ^a^	
Prostate volume, mL [IQR]		0.98 (0.97–0.99)	**0.008**	0.98 (0.97–1.00)	**0.021**
PI-RADS-2 ≥ 4 on mpMRI		1.22 (0.58–2.57)	0.599	– ^a^	

UVA—univariable analysis; MVA—multivariable analysis; OR—odds ratio; 95% CI—95-percent confidence interval; PSA—prostate-specific antigen; IQR—interquartile range; PI-RADS-2—Prostate Imaging-Reporting and Data System-2; mpMRI—multiparametric magnetic resonance imaging. ^a^ not included in the multivariable analysis. ^b^ versus grade group 1. ^c^ versus grade group 1–2. Bold: *p* < 0.05.

## Data Availability

The data analyzed in this study are available upon request from the corresponding author.
